# Endoplasmic reticulum-anchored nonstructural proteins drive human astrovirus replication organelle formation

**DOI:** 10.1371/journal.ppat.1013538

**Published:** 2025-09-22

**Authors:** Brooke Bengert, Samaneh Mehri, Madeline Holliday, Nicholas J. Lennemann

**Affiliations:** Department of Microbiology, University of Alabama at Birmingham, Birmingham, Alabama, United States of America; Heidelberg University, GERMANY

## Abstract

Human astroviruses (HAstV) are a major cause of acute, non-bacterial gastroenteritis and have been implicated in severe infections of the nervous system. Despite global prevalence, there are no established treatments for HAstVs due to a lack of understanding of the fundamental biology of infection, including mechanisms of viral replication. Like all positive-stranded RNA viruses, infection induces remodeling of host membranes into replication organelles (ROs). However, the intracellular membrane source and viral proteins involved in the coordination of HAstV ROs remain poorly defined. Using immunofluorescence microscopy, we determined that HAstV1 infection drives extensive restructuring of the endoplasmic reticulum (ER) to concentrate RNA replication and virus packaging. Long-term, time-lapse imaging of the ER and time point transmission electron microscopy (TEM) revealed that temporal manipulation of ER membrane corresponds with the emergence of ER-contiguous double membrane vesicles (DMV). The expression of transmembrane nonstructural proteins nsp1a/1, nsp1a/2, and nsp1a/1–2 led to the fragmentation of the ER for both HAstV1 and HAstV-VA1. However, only the expression of nsp1a/1–2 established DMV-like networks in the absence of an active infection. Further, super resolution microscopy revealed the organization of these two viral proteins in RO-like arrangements within the perinuclear region of infected cells. Together, these findings demonstrate the functions of nsp1a/1 and nsp1a/2 in the biogenesis of astrovirus-induced ROs, highlighting these proteins as exploitable targets for the design of antivirals restricting astrovirus replication.

## Author summary

Human astroviruses (HAstV) are understudied, globally prevalent pathogens capable of causing potentially fatal infections of the central nervous system in children, the elderly, and immunocompromised individuals. Despite the capacity to cause devastating disease, there are no established vaccines, antivirals, or therapeutics available to combat HAstV infections, in part due to a lack of knowledge for the functions of several viral proteins. This study furthers our understanding of HAstV manipulation of the host cell by identification of the viral proteins responsible for the biogenesis of virus-induced double membrane vesicles, which are a hallmark of replication.

## Introduction

The *Astroviridae* family is composed of small, non-enveloped, positive-sense RNA viruses (+ssRNA) that infect a variety of animal hosts [1,2]. Astroviruses are classified into two genera by host range, including viruses isolated from both mammals (*Mamastrovirus*) and birds (*Avastrovirus*) that cause a variety of mild to severe pathologies [2]. Human astroviruses (HAstV) belong to the *Mamastrovirus* genus and are comprised of three genetically distinct groups: the classical serotypes (HAstV1-8) and two divergent lineages, MLB and VA [1,2]. Classical astroviruses are a leading cause of non-bacterial gastroenteritis, representing 2–9% of all acute cases in children across the world and an estimated 3.9 million cases in the U.S alone every year [2,3]. Divergent human astroviruses are associated with extra-gastrointestinal and neurotropic infections in immunocompromised children, which can be fatal [4–9]. Despite the potential to cause severe disease, *Astroviridae* remains an understudied viral family of global health importance.

The ~ 6.8–7.4 kilobase (kb) astrovirus genome is comprised of three overlapping open reading frames (ORFs) that are translated as polyproteins: ORF1a, ORF1b, and ORF2 [1]. Furthermore, it has been shown that classical HAstVs and MLBs contain an alternative translation start codon within ORF2 that produces protein X, which acts as viroporin that is important for virion egress [10]. Viral nonstructural proteins (nsp) are generated from ORF1a and ORF1b, whereas structural proteins are translated from ORF2 transcripts driven by a subgenomic promoter (SGp) present near the 3’ end of ORF1b [1,11–13]. The nsp1a polyprotein (ORF1a) encodes five proposed nonstructural proteins: a single-pass transmembrane protein (nsp1a/1), a multi-pass transmembrane protein (nsp1a/2), a serine protease (nsp1a/3), a cap-like viral protein linked to the genome (nsp1a/4-VPg), and a phosphoprotein (nsp1a/4-p20) [1,14–16]. Production of the RNA-dependent RNA polymerase (RdRp, nsp1b) occurs through an infrequent (-1) ribosomal frameshift (RFS) at a conserved RNA stem loop at the junction of ORF1a and ORF1b, resulting in co-translation of the nsp1ab polyprotein [13,17,18]. While the genomic organization and predicted protein products of ORF1a and ORF1b have been defined, the specific roles of several astrovirus nonstructural proteins have yet to be characterized, which is critical for understanding the mechanisms of replication and pathogenesis [19].

Viral polyproteins are targeted to the endoplasmic reticulum (ER) by nsp1a/1, which contains a transmembrane domain that acts as a signal peptide for nsp1a/2 [20,21]. Host signal peptidase (SPase) has been proposed to mediate the cleavage between nsp1a/1 and nsp1a/2 in the ER lumen, while the remaining cytoplasmic junctions between individual nonstructural proteins are cleaved by the viral protease, nsp1a/3 [19,20,22,23]. Previous studies have reported that nsp1a/1 and nsp1a/4 colocalize with the ER and replicating RNA [21,16]. Additionally, different sequences present within the hypervariable region (HVR) of nsp1a/4 have been linked to increased virus recovered from fecal samples, suggesting that nsp1a/4 may modulate replication or pathogenicity [24]. Still, the molecular functions and interactions of nsp1a/1, nsp1a/2, and nsp1a/4 during infection remain elusive.

Like all + ssRNA viruses, astrovirus infection induces extensive rearrangements of intracellular membranes for productive replication, including the formation of double membrane vesicles (DMV) adjacent to viral particles within infected cells [25–29]. DMVs, typically derived from the ER, belong to a broad class of +ssRNA virus-induced replication organelles (ROs) [25]. These essential structures are thought to concentrate the viral RNA and replication machinery at the same topological compartment, as well as shield double-stranded RNA (dsRNA), a replication intermediate of +ssRNA viruses, from pattern recognition receptors (PRR) in the cytoplasm [25,28,30]. Among other +ssRNA viruses, membrane reorganization is mediated by viral nonstructural proteins with transmembrane or membrane-interacting domains [31–38]. However, there is a current lack of experimental evidence for the intracellular membrane source and viral proteins that drive astrovirus RO biogenesis.

In this study, we designed epitope-tagged recombinant viruses and employed a variety of complimentary microscopy techniques to characterize the extensive restructuring of ER membrane that occurs during HAstV infection. We report that dsRNA is membrane-protected, likely concentrated in the interior of ER-derived DMVs. Further, the co-expression of HAstV1 transmembrane viral proteins, nsp1a/1 and nsp1a/2, was necessary and sufficient to induce the formation of DMV-like networks in the absence of an active infection. The expression of HAstV-VA1 nsp1a/1 and nsp1a/2 was found to induce similar changes to ER morphology, indicating a conserved role for these proteins. Together, our findings establish that nsp1a/1 and nsp1a/2 function as key effectors of ER remodeling for RO biogenesis during astrovirus infection, making them ideal targets of antivirals to restrict viral replication.

## Results

### HAstV1 infection drives remodeling of ER membrane

**Fig 1:**
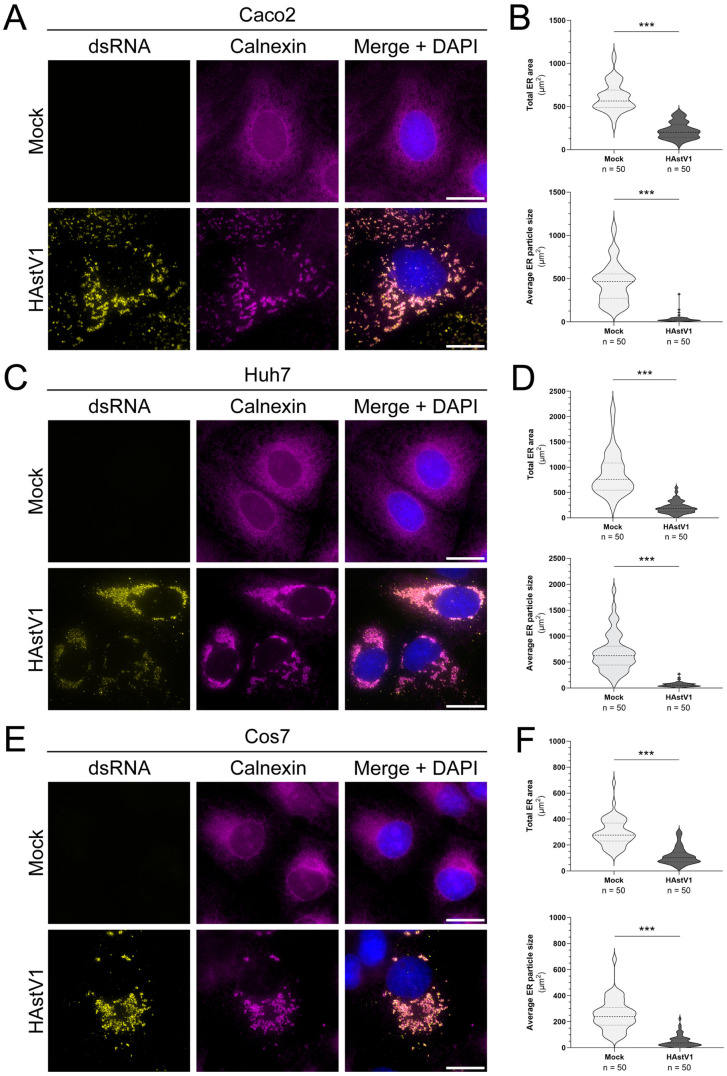
Cell type independent astrovirus ER remodeling. (A, C, E) Representative immunofluorescence staining of mock or HAstV1 infected (MOI = 3; 24 hpi) cells of the indicated type. Scale bars represent 20 µm. (B, D, F) Quantification of the total area of ER signal (top) and mean area of ER particles (bottom) between groups by particle analysis using FIJI ImageJ software and an unpaired t-test (*** p < 0.0001). Thresholding was applied uniformly between groups for n = 50 cells across 3 independent experiments. Calnexin was used as an ER marker for analysis.

Given the colocalization of nsp1a/1 and nsp1a/4 with ER membrane, it has been suggested that astrovirus ROs originate from the ER [21,16,28]. Thus, we sought to investigate morphological changes to ER membrane during HAstV1 infection using immunofluorescence (IF) microscopy. Infection of Caco2 cells resulted in aggregation of the ER in regions of dsRNA staining, which mark sites of +ssRNA virus replication (Fig 1A). Quantification of the total area and size of distinct clusters of ER marker (calnexin) signal between mock and HAstV1 infected cells revealed that infection resulted in significant ER membrane condensation and network fragmentation, respectively (Fig 1B). Furthermore, HAstV1-driven restructuring of the ER occurred in multiple cell types, as infection of two other cell lines that support HAstV1 replication (Huh7 and Cos7) produced similar aggregates of ER at 24 hours post infection (hpi) (Fig 1C and 1E). Quantification of the ER morphology in these additional cell types demonstrated the same trend of decreased ER area and particle size (Fig 1D and 1F). Thus, the consistent remodeling of the ER across multiple permissive cell lines supports an important function for ER membrane during astrovirus replication that is independent of cell type.

### Temporal restructuring of ER membrane correlates with the biogenesis of DMVs

**Fig 2:**
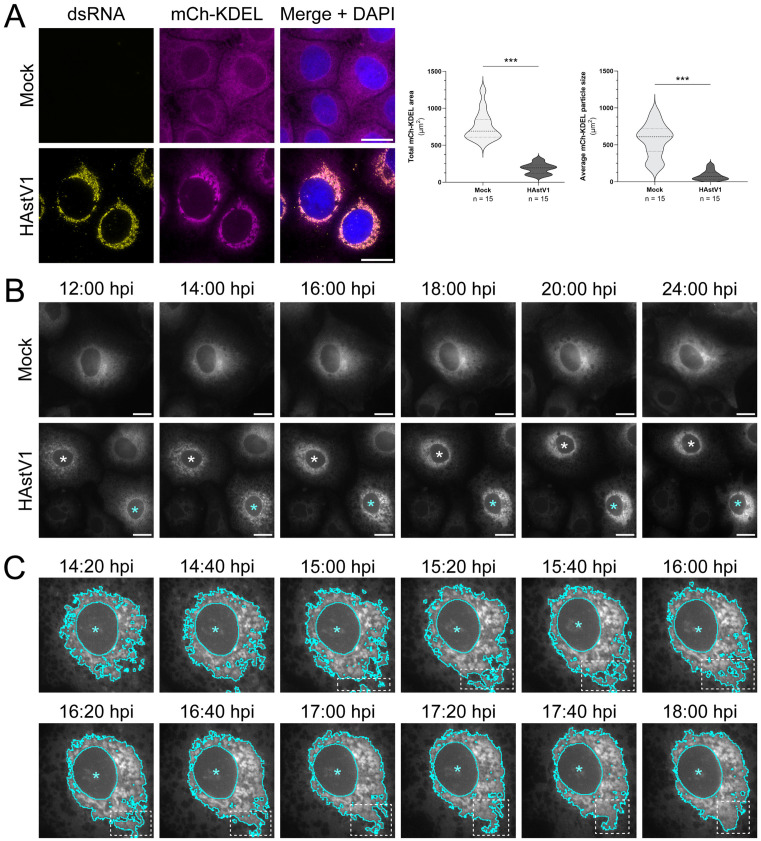
Temporal HAstV1-driven restructuring of the ER by long-term, time-lapse imaging. (A) Representative immunofluorescence staining of mock or HAstV1 infected (MOI = 3; 24 hpi) Huh7 cells expressing the mCherry-KDEL ER marker (magenta). Scale bars represent 20 µm. Quantification of the total area of ER signal (left) and mean area of ER particles (right) between groups was performed by particle analysis using FIJI ImageJ software and an unpaired t-test (*** p < 0.0001). Thresholding was applied uniformly between groups for n = 15 cells across 3 independent experiments. mCherry-KDEL was used as an ER marker for analysis. (B) Still frames from live-cell imaging of mock or HAstV1 infected (MOI = 3), mCherry-KDEL (gray) expressing Huh7 cells at the indicated times post infection. Infected cells are indicated by an asterisk (*). Scale bars represent 20 µm. (C) A higher magnification of the cell marked with a cyan asterisk from panel B, representing images taken every 20 minutes between 14:20 and 18:00 hpi. Remodeled ER is segmented in cyan. The region indicated by the white box highlights recruitment of remodeled ER to the existing aggregate.

The ER is a dynamic organelle that undergoes continuous remodeling as a part of normal cellular function and growth [39,40]. Therefore, we sought to capture temporal changes to the ER by long-term, time-lapse imaging of a fluorescent protein localized to the ER lumen, mCherry-KDEL (mCh-KDEL). Notably, fixed IF imaging of HAstV1-infected Huh7 cells resulted in similar clustering of exogenously expressed mCh-KDEL compared to endogenous calnexin staining (Figs 2A and 1B). Furthermore, live-cell imaging of mCh-KDEL in Huh7 cells demonstrated that infection-dependent ER manipulation progresses through distinct membrane intermediates throughout a 24 hour infection. Fragmentation of the ER network initiated at 12 hpi, intensified between 14–18 hpi, and culminated with the condensing of ER aggregates in the perinuclear space by 20 and 24 hpi (Figs 2B and S1 and S1 and S2 Movies). Closer examination of the dynamics of mCh-KDEL in images acquired between 14–18 hpi, the time frame between which the most dramatic changes to ER morphology were observed, confirmed that ER aggregates likely form due to recruitment of peripheral ER rather than perinuclear ER expansion (Fig 2C white boxes and S3 Movie). Indeed, we found no significant difference in the abundance of several ER resident proteins (calnexin, CLIMP63, RTN4) by immunoblot between mock and infected cell lysates at 24 hpi, confirming that ER aggregates form as a result of viral manipulation of membrane, not membrane proliferation (S2 Fig).

**Fig 3:**
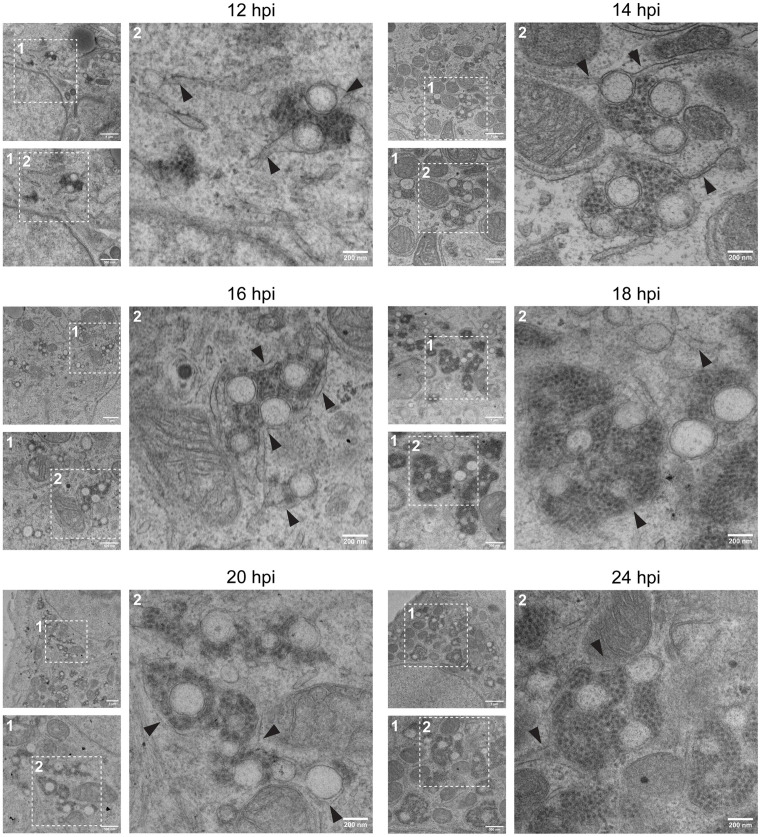
Temporal HAstV1-driven membrane alterations by transmission electron microscopy. Transmission electron microscopy (TEM) of sectioned HAstV1 infected (MOI = 3) Huh7 cells fixed at the indicated times post infection. Black arrowheads indicate ER membrane connected to DMVs. Scale bars represent the indicated distances. Insets show a higher magnification of the indicated regions.

Ultrastructural transmission electron microscopy (TEM) of HAstV1-infected cells fixed at corresponding intervals revealed that DMVs emerge and mature into virion-associated ROs throughout 12–24 hpi (Fig 3). At earlier time points (8 and 10 hpi), we did not observe assembled viral particles, appreciable DMVs, nor major alterations to ER morphology (S1 Fig). Sparse DMVs associated with *de novo* virions were first observed at 12 hpi, corresponding to early time points of detectable extracellular virus [41]. These structures increased in abundance from 14–18 hpi, and the distribution of DMVs in large, interconnected networks spanning the perinuclear space of infected cells was most evident at 20 and 24 hpi. Though some individual DMVs were observed, the double membrane of HAstV1-induced DMVs was most frequently comprised of single membrane vesicles enclosed by an ER-contiguous outer membrane (Fig 3 black arrows). Additionally, we observed clusters of newly formed virions associated with ER membrane. Together, these findings establish the biogenesis of ER-derived DMV networks as a hallmark of astrovirus infection.

### HAstV1 nonstructural proteins colocalize with remodeled ER and replicating RNA

To investigate the contributions of HAstV1 nonstructural proteins to RO formation, we generated recombinant viruses expressing epitope-tagged nonstructural proteins to enable localization studies within the context infection-induced ER remodeling. Thus, we inserted a 14-amino acid V5 epitope tag near the N- or C- terminus of each nonstructural protein using our previously published HAstV1 cDNA infectious clone (S3A Fig) [19]. Considering the sites of SPase- and viral protease-mediated cleavage, at least two amino acids were maintained adjacent to the P1 position of all cleavage junctions, and a flexible amino acid linker (GGSG) was used to adequately space the V5 tag from the remaining sequence of each nonstructural protein. Stable epitope-tagging of VPg and nsp1b was unsuccessful despite rescue of detectable rHAstV1-V5-nsp1b virus. However, recombinant viruses expressing V5-tagged nsp1a/1, nsp1a/2, nsp1a/3, and nsp1a/4 were rescued at similar titers as WT infectious clone-derived virus, suggesting that epitope tag insertion does not interfere with genome replication or packaging (S3A and S3B Figs). To validate the expression of individual V5-tagged nonstructural proteins from infection, we performed immunoblotting of cell lysates infected with each recombinant virus, all of which displayed distinct banding patterns for each expected nonstructural protein (S3C Fig). We observed bands that corresponded to the predicted sizes of V5-tagged nsp1a/1, nsp1a/2, and nsp1a/3; however, multiple low intensity bands were observed for nsp1a/4. Similar to previous studies that observed differential processing of nsp1a/4 in a polyprotein expression system, these findings support the production of intermediate proteins, which may carry out unique functions during infection [19].

**Fig 4:**
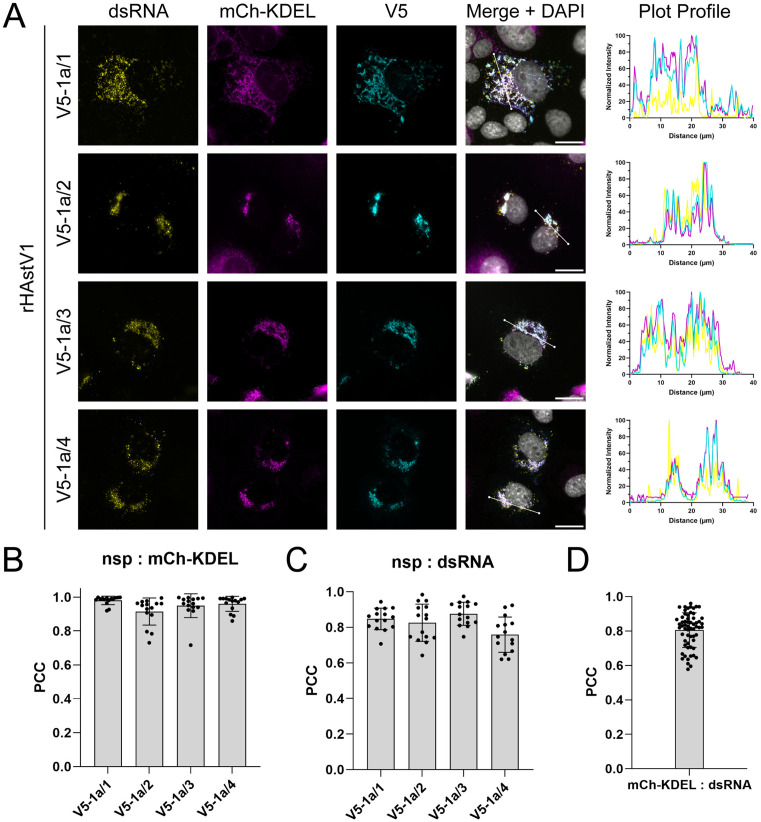
Astrovirus nonstructural proteins colocalize with dsRNA and remodeled ER. (A) Representative immunofluorescence staining of rHAstV1-V5 infected (MOI = 3; 24 hpi) Cos7 cells expressing the mCherry-KDEL ER marker. Percent maximum intensity profiles of dsRNA (yellow), mCherry-KDEL (magenta), and V5 (cyan) staining were obtained across a 40 µm linescan shown in merged images (white line) using FIJI ImageJ software. (B) Quantification of the colocalization of V5-tagged nsps with remodeled ER and (C) dsRNA. Analysis was performed by calculating the Pearson correlation coefficient (PCC) for n = 15 cells from each rHAstV1-V5 group and (D) pooling of all virus-infected groups (total n = 45) to quantify colocalization of dsRNA with remodeled ER across 3 independent experiments.

Given that ER-derived DMVs are likely sites of viral replication, we expected HAstV1 viral nonstructural proteins, which are typically involved in the replication of +ssRNA viruses, to colocalize with remodeled ER membrane. Thus, we employed our V5-tagged rHAstV1 virus panel to investigate the intracellular localization of nonstructural proteins during an active infection. Consistent with this model, all tested nonstructural proteins colocalized with ER aggregates and dsRNA (Fig 4A). Calculation of the Pearson correlation coefficient (PCC) for each group confirmed robust colocalization of all nonstructural proteins with mCh-KDEL and dsRNA (Fig 4B and 4C). In addition, the frequent overlap of dsRNA and mCh-KDEL signals further highlights the role of ER membrane in astrovirus replication (Fig 4D).

### Double-stranded RNA accumulates within the interior of DMVs

**Fig 5:**
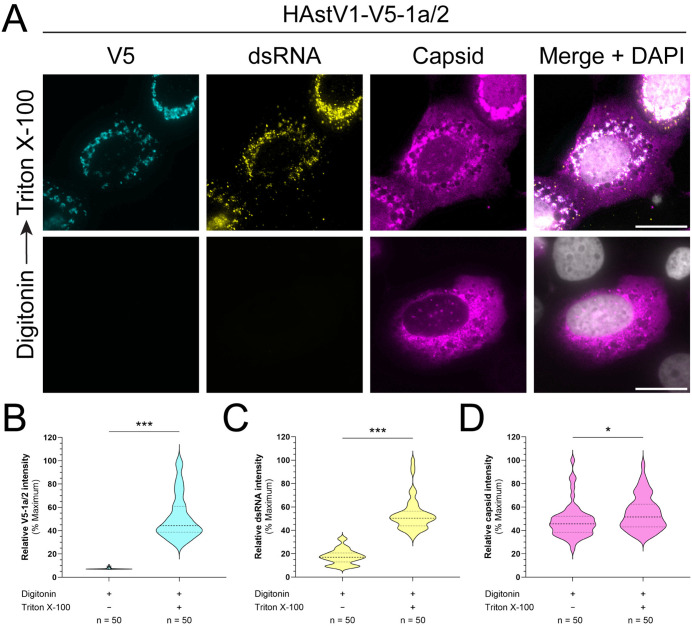
Astrovirus replication occurs within ER-derived DMVs. (A) Representative immunofluorescence staining of rHAstV1-V5-1a/2 infected (MOI = 3; 24 hpi) Huh7 cells permeabilized with digitonin alone or digitonin followed by Triton X-100. Scale bars represent 20 µm. (B) Quantification of the mean fluorescence intensity of V5 signal (cyan), (C) dsRNA signal (yellow), and (D) HAstV1 capsid signal (magenta) for n = 50 cells based on permeabilization treatment. Mean fluorescence intensity was obtained using FIJI ImageJ software from cells imaged using the same acquisition settings. Data are analyzed by an unpaired t-test and shown as a percentage of the maximum mean intensity observed across three independent experiments (* p < 0.05, *** p < 0.0001).

The interior compartments of ROs are thought to serve as a secluded niche to protect dsRNA, an immunostimulatory intermediate of +ssRNA virus replication, from detection by pattern recognition receptors in the cytoplasm [25,42,43]. Further, active RNA synthesis has been shown to occur within the ER-derived DMVs induced by several coronaviruses (CoV) [44,45]. In contrast to CoVs, some picornaviruses, which are non-enveloped like HAstVs, assemble their replication complexes on the cytoplasmic face of ROs [46]. Given the similar architecture of CoV and astrovirus ROs, these differences raise the question of the membrane orientation of HAstV replication. To elucidate the site of dsRNA accumulation during HAstV1 infection, we employed a differential permeabilization approach to immunolabel dsRNA in cells infected with our recombinant HAstV1-V5-nsp1a/2 virus, which encodes an ER lumen-localized V5 tag. Selective permeabilization of the plasma membrane with a low concentration of digitonin did not permit antibody-mediated staining of the membrane-protected V5 tag nor dsRNA, whereas complete permeabilization with Triton X-100 rendered both epitopes detectable (Fig 5A, 5B, and 5C). In contrast, staining of HAstV1 capsid was observed in both conditions, confirming that cytoplasmic epitopes were accessible upon digitonin treatment; however, full permeabilization with Triton X-100 exhibited an increased average capsid intensity over digitonin treated groups (Fig 5A and 5D). These results indicate that dsRNA is stored within a membrane-protected compartment during HAstV1 infection, likely the interior of DMVs.

### Nsp1a/1 and nsp1a/2 drive ER remodeling for DMV biogenesis

**Fig 6:**
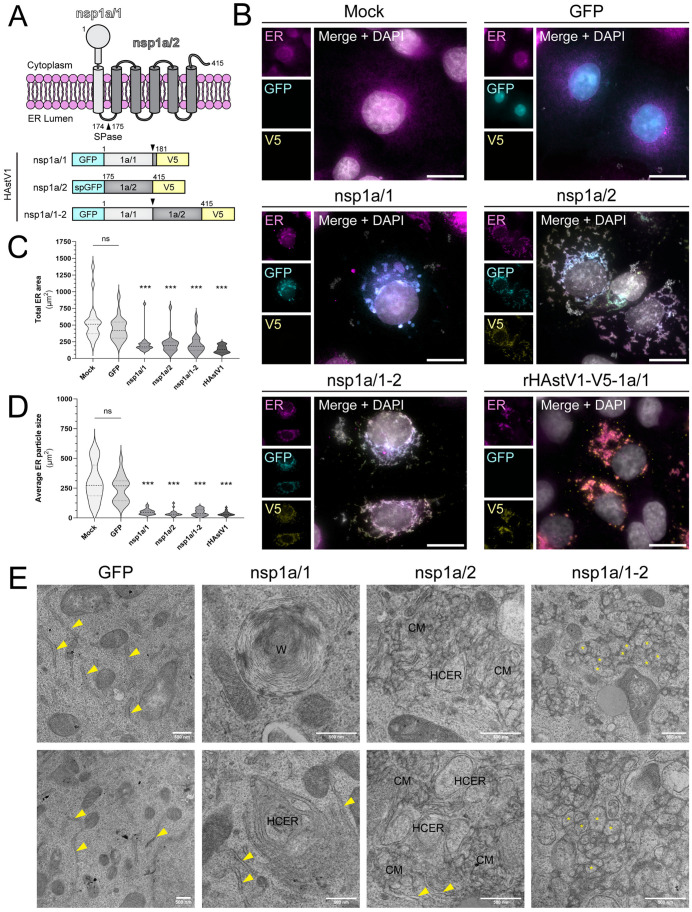
Nsp1a/1 and nsp1a/2 are the minimal constituents for HAstV1 DMV biogenesis. (A) Schematic of nsp1a/1 and nsp1a/2 membrane topology and construction of dual-tagged HAstV1 expression plasmids. Nsp1a/1 (light gray), nsp1a/2 (dark gray). Black arrowheads represent sites cleaved by host signal peptidase (SPase). Numbers indicate HAstV1 amino acid positions. (B) Representative immunofluorescence staining of Cos7 cells transfected with the indicated constructs or infected with rHAstV1-V5-1a/1 (MOI = 3) at 24 hours post treatment. Scale bars represent 20 µm. (C) Quantification of the total area of ER signal and (D) mean area of ER particles between groups by particle analysis using FIJI ImageJ software and one-way ANOVA with multiple comparisons versus mock treated cells (*** p < 0.0001). Thresholding was applied uniformly between groups for n = 20 cells across 3 independent experiments. Calnexin was used as an ER marker. (E) Transmission electron microscopy (TEM) of sectioned Cos7 cells transfected with the indicated constructs and fixed at 24 hours post transfection. Yellow arrowheads highlight typical ER membrane morphology. ER whorl (W), convoluted membranes (CM), high curvature ER (HCER). Yellow asterisks (*) indicate representative vesicles with a double membrane component. Scale bars represent 500 µm.

Among +ssRNA viruses, membrane reorganization is mediated by transmembrane-domain containing nonstructural proteins, where all or the majority of these virus-encoded membrane-interacting proteins coordinate RO biogenesis [25,30]. Notably, astroviruses encode only two nonstructural proteins with transmembrane domains: nsp1a/1 and nsp1a/2. Therefore, we hypothesized that these two viral proteins function as essential drivers of HAstV-induced ER remodeling. To study the independent roles of these proteins, we generated dual-tagged expression plasmids that encode HAstV1 nsp1a/1, nsp1a/2, or both (nsp1a/1–2) with an N-terminal green fluorescent protein (GFP) fusion and a C-terminal V5-epitope tag (Fig 6A). To maintain nsp1a/2 topology, a BiP signal peptide (sp) sequence was added to the N-terminus of GFP (spGFP) to correctly localize the N-terminus of nsp1a/2 to the ER lumen. As expected, expression of either viral protein in Cos7 cells induced ER remodeling relative to mock and GFP-transfected controls (Fig 6B). Expression of nsp1a/1 formed circular ER fragments, whereas nsp1a/2 expression resulted in severe fragmentation of the ER network spanning the entire cell (Fig 6B). The expression of nsp1a/1–2 induced ER alterations that most resembled infection; however, quantification of the total area and size of distinct clusters of ER between groups did not resolve these qualitative differences (Fig 6C and 6D).

Further evaluation of cellular ultrastructure by TEM confirmed that single expression of each protein resulted in unique membrane alterations, whereas GFP transfection alone did not alter ER morphology (Fig 6E). We observed the presence of altered membranes in 9 out of 12 imaged nsp1a/1-transfected cells, where 4 contained large ER whorls (W) and 5 contained high-curvature ER (HCER) clusters. In contrast, networks of HCER associated with convoluted membranes (CM) were present in nsp1a/2-expressing cells. However, only the co-expression of nsp1a/1 and nsp1a/2 resulted in the emergence of DMV-like structures resembling infection-induced ROs (Fig 6E). These nsp1a/1–2-induced DMVs appeared to be tightly packed, likely due to the absence of RNA replication and virion production in our protein expression system. Furthermore, like infection-induced structures, the vesicles formed from expression of nsp1a/1–2 were linked together by a contiguous outer membrane, forming an expansive network of ER-derived structures reminiscent of infection-induced DMV networks. Together, these findings support a model in which nsp1a/1 and nsp1a/2 are independently capable of ER manipulation, but the coordinated activity between the two is required to establish a functional replication organelle.

### HAstV-VA1 nsp1a/1 and nsp1a/2 remodel the ER

**Fig 7:**
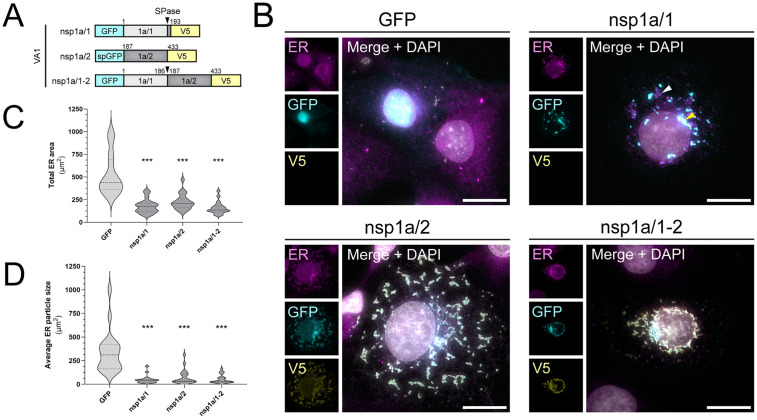
Conservation of nsp1a/1- and nsp1a/2-mediated ER remodeling for HAstV-VA1. (A) Construction of dual-tagged HAstV-VA1 expression plasmids. Black arrowheads represent sites cleaved by host signal peptidase (SPase). Numbers indicate VA1 amino acid positions. (B) Representative immunofluorescence staining of Cos7 cells transfected with the indicated constructs at 24 hours post transfection. Scale bars represent 20 µm. Arrowheads indicate regions of over- (yellow) or undersaturation (white) during imaging of nsp1a/1. (C) Quantification of the total area of ER signal and (D) mean area of ER particles between groups by particle analysis using FIJI ImageJ software and one-way ANOVA with multiple comparisons versus GFP-transfected cells (*** p < 0.0001). Thresholding was applied uniformly between groups for n = 20 cells across 3 independent experiments. Calnexin was used as an ER marker.

To investigate whether the functions of nsp1a/1 and nsp1a/2 may be conserved among other mamastroviruses (MAstV), we aligned the sequences of 10 MAstV isolates. Pairwise sequence alignment revealed a low abundance of completely conserved residues, particularly between classical HAstV1 (MA01) and divergent VA1 strains (MA09), which demonstrated percent identity scores of 23.5% and 21.3% for nsp1a/1 and nsp1a/2, respectively (S4A and S4B Fig, asterisks). However, it has been shown by TEM that VA1 infection, like HAstV1, leads to the formation of DMVs, suggesting that nsp1a/1 and nsp1a/2 may provide conserved functions in astrovirus RO biogenesis [29]. Thus, despite a low sequence conservation, we hypothesized that VA1 nsp1a/1 and nsp1a/2 would demonstrate similar ER remodeling capacities as HAstV1 proteins. To investigate the ER alterations produced by VA1 nsp1a/1, nsp1a/2, and nsp1a/1–2, we generated the same dual-tagged expression plasmids encoding proteins cloned from VA1 (Figs 6A and 7A). Indeed, we found that expression of either viral protein alone or in tandem induced quantitative ER remodeling relative to a GFP-transfected control (Fig 7C and 7D). During imaging of the nsp1a/1 construct, some regions displayed extreme GFP-nsp1a/1 intensities that were oversaturated even at low exposure times (yellow arrowheads), which resulted in underexposure of remaining GFP signal (white arrowheads) (Fig 7B). Despite this, we observed membrane alterations for nsp1a/1, nsp1a/2, and nsp1a/1–2 that resembled those induced by HAstV1 viral proteins (Figs 6B and 7B). Thus, our results demonstrate a functionally conserved role for astrovirus nsp1a/1 and nsp1a/2 in the manipulation of ER membrane for RO biogenesis among both classical and divergent HAstVs.

### Super resolution dSTORM identifies nsp1a/1 and nsp1a/2 at DMVs

**Fig 8:**
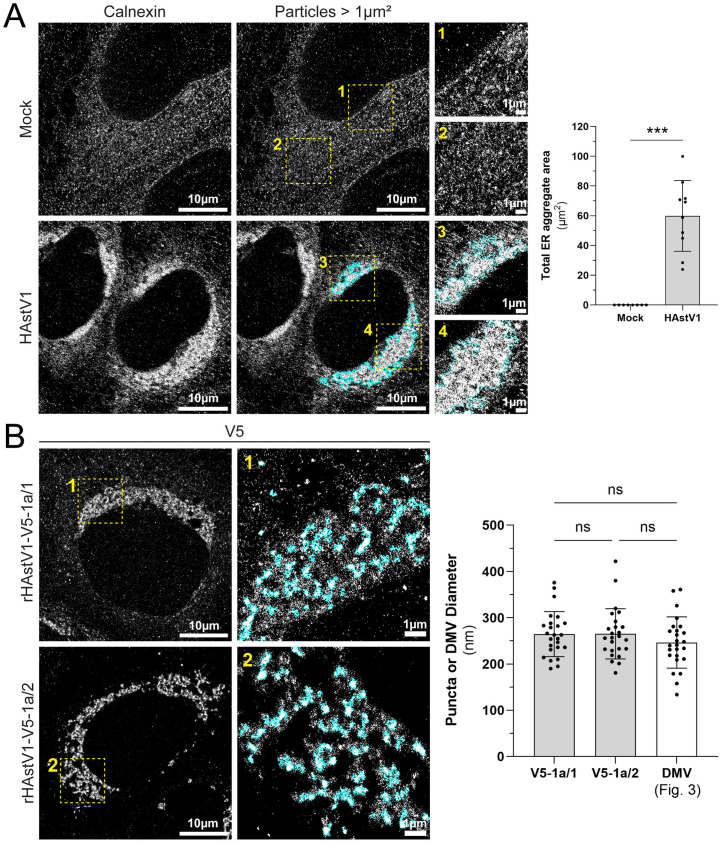
Remodeled ER architecture by super resolution dSTORM. (A) Representative dSTORM images of immunolabeled calnexin in mock or HAstV1 infected (MOI = 3; 24 hpi) Huh7 cells. Segmented particles greater than 1 µm^2^ within a single cell region of interest (ROI) are highlighted in cyan. Insets (1-4) show a higher magnification of the indicated regions. Scale bars represent the indicated distances. Quantification of the total area of dense ER particles in mock and HAstV1 infected cells was performed by thresholding and particle analysis using FIJI ImageJ software and an unpaired t-test (*** p < 0.0001). (B) Representative dSTORM images of immunolabeled V5 in rHAstV1-V5-1a/1 and -1a/2 infected (MOI = 3; 24 hpi) Huh7 cells. Insets (1-2) show a higher magnification of the indicated regions. Scale bars represent the indicated distances. Representative particles used for V5 puncta diameter analysis are segmented in cyan. Quantification of the average V5 puncta diameter from dSTORM images and DMV diameter from TEM micrographs shown in Fig 3 (24 hpi) was performed using the measure tool in FIJI ImageJ software and a one-way ANOVA with multiple comparisons between all groups (ns, not significant).

Due to limited spatial resolution, diffraction-limited epifluorescence microscopy is poorly suited to resolve crowded cellular structures. To overcome this limitation, we employed direct stochastic optical reconstruction microscopy (dSTORM) to investigate the nanoscale localization of nsp1a/1 and nsp1a/2 using our V5-tagged recombinant HAstV1 viruses. Infected Huh7 cells displayed large perinuclear ER aggregates captured by particle analysis of clusters exceeding 1 μm^2^, which were absent in all mock-treated cells (Fig 8A). Furthermore, nsp1a/1 and nsp1a/2 were localized in similar perinuclear aggregates (Fig 8B). Notably, the increased precision of V5 localization by dSTORM afforded resolution of both nsp1a/1 and nsp1a/2 organized in discrete puncta with an average diameter of 250 nm (Fig 8B). These structures closely resemble the size and clustered distribution of DMVs visualized by TEM, which we observed to average 250 nm in diameter at 24 hpi, suggesting that nsp1a/1 and nsp1a/2 localize to DMV membranes during HAstV1 infection (Figs 3 and 8B) [28]. Together, these data support the synergistic roles of transmembrane nonstructural proteins nsp1a/1 and nsp1a/2 as membrane-bound components and functional drivers of DMV biogenesis.

## Discussion

In this study, we evaluated the remodeling of the ER membrane driven by viral nsp1a/1 and nsp1a/2. In a protein overexpression system, we established that expression of nsp1a/1–2 induced DMV-like vesicles within dilated ER membrane (Fig 6E). Though these structures were more compact and demonstrated a larger intralumenal space than infection-induced DMVs, we attribute these differences to a lack of replicating RNA and assembling viral particles, as well as an excess accumulation of protein in these membranes due to the overexpression system. Overall, our results indicate that nsp1a/1 and nsp1a/2 act synergistically to drive the remodeling of ER membrane for the formation of large DMV networks that support viral RNA replication and virion production during astrovirus infection.

Virus-induced membrane alterations are thought to serve as isolated environments that shield dsRNA and viral RNA from host immune surveillance [25,42,43]. Nevertheless, some non-enveloped RNA viruses replicate in association with the cytoplasmic surface of ROs [46]. To elucidate the site of dsRNA accumulation for HAstV1 ROs, we performed IF staining using a low concentration of digitonin to selectively permeabilize the plasma membrane. Using this strategy, we determined that dsRNA is not exposed to the cytosol, suggesting that HAstV1 replication is confined to the interior of infection-induced DMVs (Fig 5). However, the accumulation of dsRNA within DMVs does not equate active RNA synthesis. Further study is necessary to establish the precise site of active astrovirus replication in relation to DMVs. For viruses that replicate within the interior of ROs, the translation of viral proteins occurs in a topologically separate environment from replication. Thus, these viruses devise specialized strategies to allow +ssRNA to reach the cytoplasm for translation and packaging of viral genomes into progeny viral particles. Though the exact membrane orientation of HCV replication is unclear, both HCV and orthoflavivirus ROs have been observed to contain small openings to the cytoplasm that could accommodate the release of genomic RNA [47,48]. However, the existence of viral or host proteins regulating this process has not been characterized. In contrast, recent studies have identified a CoV pore complex consisting of nsp3 and nsp4 that bridges the DMV interior to the cytoplasm, enabling viral RNA export and capture by nucleocapsid proteins [49–51]. Given the potential interior membrane orientation of HAstV replication, it is possible that astroviruses may employ a similar strategy to manage translation and virion assembly. Future studies leveraging structural biology and reverse genetics approaches may uncover the mechanisms that govern astrovirus RNA localization for the continued translation of +ssRNA and packaging of viral genomes.

In this study, we identified that nsp1a/1 and nsp1a/2 function as drivers of RO biogenesis and structural components of DMV membranes. The expression of nsp1a/1 and nsp1a/2 individually induced extensive restructuring of the ER, but the presence of both viral proteins was required to form DMV-like networks (Fig 6). However, the underlying contributions of these proteins to RO structure and function remain elusive. Nsp1a/1 contains a single transmembrane domain that also functions as a signal peptide to direct translation of viral polyproteins to the ER [1,21]. As such, nsp1a/1 is removed from the N-terminus of polyproteins through a highly efficient cleavage event performed by a host signal peptidase (S3C Fig) [20,21]. Previous studies demonstrated that conserved di-arginine motifs present within nsp1a/1 contribute to the perinuclear retention of this viral protein, as alanine substitution of these motifs resulted in diffuse, reticular staining of nsp1a/1 [21]. Consistent with these findings, we observed nsp1a/1 localized to clustered ER membrane (Fig 4A). However, further investigation of infected cells by super resolution dSTORM revealed the presence of a small proportion of nsp1a/1, but not nsp1a/2, dispersed in a reticular network that was not resolvable by traditional epifluorescence imaging (Fig 7B). Similar to observations made by IF and live-cell imaging, there was often weak staining of ER membrane in the periphery of infected cells at 24 hpi (Figs 1 and 2). Together, these data suggest that diffusely localized nsp1a/1 may contribute to the recruitment of ER membrane to perinuclear aggregates, perhaps by coordination of di-arginine motifs, or by an otherwise uncharacterized mechanism.

Ectopic expression of nsp1a/1 resulted in the accumulation of large ER whorls observed by TEM, structures that have been characterized previously to form in response to ER stress (Fig 6E) [52]. Thus, it remains unclear whether the contribution of nsp1a/1 to DMV biogenesis is by direct manipulation of ER membrane or by the induction of ER stress, which could be accomplished by interaction with ER resident host proteins. Of note, similar concentric membrane structures have been observed upon expression of transmembrane nonstructural proteins from other viral families, such as MERS-CoV [53].

Though the specific membrane topology of nsp1a/2 is poorly understood, it has been predicted to contain 4–6 transmembrane domains [1]. Given the ER-lumen localized N-terminus, we speculate that nsp1a/2 encodes 5 transmembrane domains, as this orientation would permit cytoplasmic localization of the C-terminus that is cleaved by the viral protease (Fig 6A) [19]. However, the functional significance of nsp1a/2 during infection remains elusive. In this report, we demonstrated that expression of nsp1a/2 induces substantial fragmentation of the ER network and the formation of extensively convoluted ER membrane (Fig 6). In contrast to nsp1a/1 localization, dSTORM of cells infected with a V5-nsp1a/2 encoding virus revealed that nsp1a/2 localizes exclusively to perinuclear puncta that exhibit the same average diameter as DMVs, suggesting that its role is specific to DMVs and the membranes it alters for DMV biogenesis (Fig 7B). Therefore, our recombinant HAstV1-V5-1a/2 virus may serve as a useful marker for DMV membranes in future mechanistic studies. Among different +ssRNA viral families, highly hydrophobic, multi-pass transmembrane proteins are commonly involved in the direct transformation of membrane during virus-induced RO formation [25,47,48,53–64]. Accordingly, we have now established nsp1a/2 as a previously unrecognized determinant of ER-derived RO biogenesis during astrovirus infection.

Collectively, our study revealed that restructuring of ER membrane into DMV networks is a defining feature of productive astrovirus infection. We identified the two viral proteins responsible for ER manipulation for DMV formation: nsp1a/1 and nsp1a/2. Further, we validated the conserved functions of these two viral proteins sourced from both a classical (HAstV1) and divergent (VA1) astrovirus lineage. Thus, future studies will be directed at elucidating the domains of nsp1a/1 and nsp1a/2 responsible for astrovirus RO formation. Together, these findings will provide insights into the rational design of nsp1a/1- or nsp1a/2-targeted inhibitors, which could serve as effective antivirals to limit astrovirus replication.

## Materials and methods

### Cell culture

Caco2 cells (ATCC, HTB-37) were maintained in modified Eagle’s medium supplemented with 20% FBS, 10% sodium pyruvate, 10% non-essential amino acids, and 100 IU penicillin per 100 μg/mL streptomycin. Human hepatoma cells (Huh7) were maintained in Dulbecco’s modified Eagle’s medium supplemented with 10% FBS and 100 IU penicillin per 100 μg/mL streptomycin and 9 g/L glucose. Cos7 cells (a gift from Dr. Alexa Mattheyses, University of Alabama at Birmingham) and HEK293T cells (ATCC, CRL-11268) were maintained in Dulbecco’s modified Eagle’s medium supplemented with 10% FBS and 100 IU penicillin per 100 μg/mL streptomycin. Hybridoma cell lines (ATCC, CRL-8795) that were used to generate monoclonal antibody 8E7 (mouse anti-HAstV1 capsid protein) were grown in Iscove’s modified Dulbecco’s medium with 2mM L-glutamine and 15% heat inactivated fetal bovine serum. All cells were cultured at 37°C and 5% CO_2_.

### Preparation of virus stocks

Virus was recovered by transfection of Huh7 cells with pcDNA-HAstV1 or pcDNA-HAstV1-V5 plasmids using polyethylenimine (PEI, 25 kDa) at a 1:1 ratio of DNA (μg) to 1 mg/mL PEI stock. At 12 hours post transfection, cells were gently washed with PBS, and the media was replaced with serum-free media containing 10 μg/mL porcine trypsin. At 72 hours post transfection, cells were lysed in their supernatants by three subsequent freezes in liquid nitrogen and thaws at 37°C. Cell debris was removed by centrifugation at 5,000 x g for 5 minutes, then virus-containing supernatants were collected and stored at -80°C until use.

### Virus titration

Caco2 cells were seeded in a 96-well plate at 2.5 × 10^4^ cells/well and allowed to grow for 72 hours to reach confluence. Media was replaced with serum-free Caco2 media supplemented with 0.3% bovine serum albumin (BSA) for 1 hour, followed by infection with 10-fold dilutions of virus-containing supernatant. At 20 hours post infection, cells were fixed in PBS + 4% paraformaldehyde (PFA), permeabilized with PBS + 0.1% Triton X-100, then washed with PBS. Primary incubation with mouse anti-HAstV1 capsid protein (8E7) diluted 1:4 in PBS was performed overnight at 4°C. Following primary, cells were washed with PBS, then incubated with goat anti-mouse Alexa Fluor 488-conjugated secondary antibody diluted 1:1000 in PBS for 30 minutes, followed by two final PBS washes. Capsid positive foci were counted in technical duplicate using an Olympus IX83 inverted fluorescence microscope to calculate titer (FFU/mL).

### Immunofluorescence microscopy

Cells were grown in 8-well chamber slides (Celltreat), fixed in PBS + 4% paraformaldehyde (PFA) for 10 minutes, permeabilized with PBS + 0.1% Triton X-100 for 10 minutes, then washed with PBS. Primary incubation was performed overnight at 4°C with antibodies diluted in PBS. Primary antibodies used for immunofluorescence staining were as follows: rabbit anti-Calnexin (Abcam, ab22595, 1:1000), mouse anti-dsRNA (GeneTex, GTX641519, 1:500), mouse anti-V5 (Invitrogen, 46–0705, 1:5000), rabbit anti-V5 (Cell Signaling, 13202, 1:1000), and mouse anti-HAstV1 capsid (8E7, 1:4). Following primary incubation, cells were washed with PBS, then incubated with corresponding Alexa Fluor 488, 594, or 647-conjugated secondary antibody (Invitrogen) diluted 1:1000 in PBS for 30 minutes, followed by a PBS wash. Cells were incubated in 300nM 4’,6-diamidino-2-phenylindole (DAPI) for 5 minutes, followed by two final PBS washes. Glass slides were mounted using a 22 x 50 mm glass coverslip (FisherScientific) and ProLong Diamond Antifade Mountant (Invitrogen, P36965), then allowed to cure overnight at room temperature. Epifluorescence images were acquired using an Olympus IX83 inverted fluorescence microscope using a 60x oil objective.

### Digitonin selective permeabilization assays

Cells grown in 8-well chamber slides (Celltreat) were incubated on ice for 5 minutes, washed with Hank’s Balanced Salt Solution (HBSS), and treated with HBSS + 25 µg/mL digitonin on ice for 5 minutes. Following digitonin incubation, cells were again washed with HBSS, then fixed in PBS + 4% paraformaldehyde (PFA) for 10 minutes at room temperature. Cells receiving subsequent full permeabilization were incubated in PBS + 0.1% Triton X-100 for 10 minutes, whereas selective permeabilization groups were incubated in PBS only. Immunofluorescence staining was performed as described. For analysis of mean fluorescence intensity between digitonin and fully permeabilized cells, all images across three independent replicates were acquired using an Olympus IX83 inverted fluorescence microscope with the same exposure settings. Mean fluorescence intensity from raw images was captured in single-cell regions of interest, then transformed as a percentage of the maximum signal.

### Immunoblots

Infected Caco2 cells were lysed in a 1X RIPA + protease inhibitor cocktail (Sigma) at 24 hours post infection. Lysates were clarified by centrifugation at 12,000 x g and 4°C for 10 minutes, then prepared with a 6X SDS loading buffer + betamercaptoethanol (BME). Prepared lysates were separated by SDS-PAGE on a 4–20% Tris-glycine polyacrylamide pre-cast gel (BioRad) for 50 minutes, followed by transfer to nitrocellulose membrane for 50 minutes. Membranes were blocked for 30 minutes in PBS + 10% non-fat milk, then probed using primary antibodies diluted in PBST + 5% BSA. Primary antibodies used include mouse anti-V5 (Invitrogen, 1:5000), rabbit anti-GAPDH (ProteinTech, 1:2000), rabbit anti-Calnexin (GeneTex, 1:5000), rabbit anti-CKAP4/CLIMP63 (ProteinTech, 1:5000), rabbit anti-RTN4 (ProteinTech: 1:5000). Proteins were observed using near-infrared dye-conjugated secondary antibodies (LiCor) diluted in PBST + 5% non-fat milk, then imaged on a LiCor Odyssey CLx imaging system.

### Plasmids

All pcDNA-HAstV1-V5 tagged infectious clone plasmids were cloned by assembly of 5’ and 3’ PCR fragments into the PstI and AgeI restriction sites of pcDNA-HAstV1 [19]. For all reactions, the vector was generated by digesting pcDNA-HAstV1 with restriction enzymes PstI and AgeI (New England BioLabs). The 5’ PCR fragment was generated using Q5 High Fidelity DNA polymerase (New England BioLabs) with AstV-PstI_F and a site-specific reverse primer. Site specific reverse primers used were V5-1a/1_R, V5-1a/2_R, V5-1a/3_R, and V5-1a/4_R (S1 Table). The 3’ PCR fragment was generated with a site-specific forward primer and AstV-AgeI_R. Site-specific forward primers used were V5-1a/1_F, V5-1a/2_F, V5-1a/3_F, and V5-1a/4_F (S1 Table).

The generation of pcDNA-GFP-V5, used as a vector for assembly of dual-tagged viral protein expression plasmids, was performed as previously described [19]. The pcDNA-GFP-V5 plasmid was linearized with BamHI (New England BioLabs) and assembled with DNA encoding HAstV1 or VA1 viral proteins. DNA encoding HAstV1 viral proteins was amplified from pcDNA-HAstV1. DNA encoding VA1 viral proteins was amplified from cDNA generated using SuperScript III Reverse Transcriptase and poly-dT primer (IDT) from viral RNA extracted from stock virus (a gift from Dr. Stacy Schultz-Cherry, St. Jude Children’s Research Hospital) using PureLink Viral RNA/DNA Mini Kit (Invitrogen). PCR products for the generation of GFP-1a/1-V5, GFP-1a/2-V5, and GFP-1a/1–2-V5 constructs for HAstV1 and VA1 were amplified using Q5 High Fidelity DNA polymerase and the following respective primers (denoted HAstV1- or VA1-): 1a/1_F with 1a/1_R, 1a/2_F with 1a/2_R, and 1a/1_F with 1a/2_R (S1 Table). Generation of spGFP-1a/2-V5 for HAstV1 and VA1 was accomplished by assembly of pcDNA-GFP-V5 digested with BamHI and MluI (New England BioLabs) with two PCR fragments amplified from the respective viral GFP-1a/2-V5 plasmid using the following respective primers: MluI-CMV_F with SpBIP-GFP_R, and SpBIP-GFP_F with 1a/2_R (S1 Table).

All PCR products and vectors were purified by agarose gel electrophoresis and extracted with Quick Gel extraction kit (Invitrogen, K220010). Plasmids were assembled by HiFi Assembly (New England BioLabs), and reactions were transformed into Stable Competent *E. coli* (New England BioLabs, C3040I). HAstV1 protein expression plasmids and infectious clones were grown for 20 hours at 30°C. VA1 protein expression plasmids were grown at 25°C for 48 hours to promote stability in *E. coli.* Plasmids were purified by ZymoPURE Express Plasmid Midiprep Kit according to the manufacturer’s protocol, then sequenced using Plasmidsaurus.

### Transfections and lentivirus production

Cos7 cells were transfected using TransIT LT-1 transfection reagent (Mirus Bio) according to the manufacturer’s protocol. For generation of mCherry-KDEL lentivirus, HEK293T cells were transfected with 1 µg pLJM1-mCherry-KDEL, 0.75 µg psPAX (Addgene plasmid #12260), and 0.25 µg pCAGGS-G-Kan (a gift from Todd Green, University of Alabama at Birmingham) using polyethylenimine (PEI, 25 kDa) at a 1:1 ratio of DNA (μg) to 1 mg/mL PEI stock. Cells were incubated at 37°C for 48 hours, then cell supernatant was harvested. Debris was pelleted by centrifugation at 300 x g for 5 minutes, then lentivirus-containing supernatants were collected and stored at -80°C until further use.

### Live-cell imaging

Huh7 cells were transduced with pLJM1-mCherry-KDEL lentivirus at 1 × 10^4^ cells/well in an 8-well polymer live imaging slide (Ibidi), then allowed to grow for 48 hours. Media was removed and replaced with virus diluted in serum free media. After absorption for 1 hour at 37°C, well volume was brought up to 200 μL with serum-containing media. Live-cell imaging was performed using an Olympus IX83 inverted fluorescence microscope in a humidified chamber at 37°C and 5% CO_2_. Epifluorescence images were acquired every 20 minutes for 20 hours using the autofocus function and a 40x air objective. Final images at 24 hpi were acquired manually using the same exposure settings.

### Transmission electron microscopy

Cos7 and Huh7 cells were seeded at 4 × 10^5^ cells/well in a 6-well plate and allowed to adhere for 24 hours prior to transfection or infection. Cells were fixed using 3% glutaraldehyde in 0.1M cacodylate buffer for 1 hour, then scraped and pelleted at 300 x g for 5 minutes to preserve ultrastructure. Carbon TEM grids overlaid with thin slices of sectioned cell pellets were prepared at the University of Alabama at Birmingham High Resolution Imaging Facility. Imaging was performed using a JOEL 1400 HC Flash TEM at 120 kV with an AMT NanoSprint43 Mk-II CMOS camera.

### Multiple sequence alignment

SignalP6.0 (DTU Health Tech) was used to determine the signal peptidase cleavage site between nsp1a/1 and nsp1a/2 for 10 different mamastroviruses (AZB52190.1, YP_009380530.1, YP_009094278.2, QPD02149.1, AWI14299.1, AFD61562.1, ACX85471.1, YP_003090286.1, UYW66761.1, NP_059945.2) [65]. These sequences were then aligned using MUSCLE (EMLB-EBI) and visualized with Jalview [66]. Pairwise alignments between HAstV1 and HAstV-VA1 were performed with Jalview to determine the percent identity.

### Super resolution dSTORM

Immunofluorescence cell samples were prepared as described in an 8-well glass bottom live-imaging slide (Ibidi) using Alexa Fluor 647-conjuagated secondary antibodies. Imaging was performed in a photoswitching buffer containing 50mM Tris-HCl and 10mM NaCl supplemented with 10% (w/v) glucose, 2% catalase and glucose oxidase solution (GLOX) and 50mM cysteamine (MEA) (a gift from Dr. Alexa Mattheyses, University of Alabama at Birmingham). dSTORM images were obtained using a Nikon Ti-2 microscope using a 100X 1.49 NA oil immersion objective and 647 nm excitation laser at the University of Alabama at Birmingham High Resolution Imaging Facility. For each image, 10,000 frames were acquired and reconstructed in Nikon Elements.

### ER morphology analysis

For analysis of epifluorescence ER morphology, signal overlapping with DAPI was excluded to eliminate ER signal corresponding to out of focus light from thick cells. The signal intensity histogram was scaled to fit values 0–255, and thresholding was performed under the same conditions between mock and HAstV1 infected cells to capture signal for particle analysis in single-cell regions of interest (ROI) drawn using the freehand selection tool. Particles less than 2 µm^2^ were excluded due the presence of pixelated edges from thresholding. For analysis of dSTORM ER morphology, reconstructed images were exported at 4 nm per pixel and subjected to Gaussian blur preprocessing (σ = 2) to merge closely spaced pixels within dense localization clusters. Thresholding was performed with no exclusion criteria to capture total signal for particle size analysis in single-cell ROI. The total area of particles greater than 1 µm^2^ was measured to quantify regions of dense ER aggregation and exclude typical reticular staining.

### Figures and statistical analysis

Figures were generated using Adobe Illustrator. Image analysis was performed using FIJI ImageJ software with statistical analysis in GraphPad Prism 10.

## Supporting information

S1 Fig Appreciable DMV biogenesis does not initiate prior to 10 hpi. (A) Still frames from live-cell imaging of mock or HAstV1 infected (MOI = 3) Huh7 cells expressing the mCherry-KDEL ER marker (gray) at 8 and 10 hours post infection (hpi). Infected cells are indicated by an asterisk (*), with cyan indicating the cell highlighted in Fig 2C and S3 Movie. Scale bars represent 20 µm. (B) Transmission electron microscopy (TEM) of sectioned HAstV1 infected (MOI = 3) Huh7 cells fixed at 8 and 10 hpi. Scale bars represent 1 µm.
(TIF)

S2 Fig HAstV-driven ER remodeling is independent of ER proliferation. Immunoblot of mock or rHAstV1-V5-1a/1 infected (MOI = 0.25, 0.5, 1, 3) Caco2 cells lysed at 24 hpi. Immunoblots were probed for ER resident proteins (calnexin, CLIMP63, RTN4), V5, and GAPDH.
(TIF)

S3 Fig Construction and rescue of V5-tagged recombinant HAstV1s. (A) Schematic of the pcDNA-HAstV1 infectious clone with V5-epitope tag locations within nsp1a/1 (lime), nsp1a/2 (green), nsp1a/3 (teal), and nsp1a/4 (blue) indicated by black rectangles with the specific inserted sequences and amino acid positions displayed below the tag. The P1 position of junctions targeted for cleavage by host signal peptidase (SPase, black) and nsp1a/3 (teal) is shown as arrowheads with the indicated P1 amino acid positions. (B) Titers of WT and rHAstV1-V5 viruses recovered from pcDNA-HAstV1 transfected Huh7 cells. Data are shown as the average ± SD focus-forming units per milliliter (FFU/mL) of supernatants serially diluted on Caco2 cells. (C) Immunoblot of lysates from WT or rHAstV1-V5 infected (MOI = 3; 24hpi) Caco2 cells. Immunoblots were probed for V5 and GAPDH.
(TIF)

S4 Fig Mammalian astrovirus nsp1a/1 and nsp1a/2 multiple sequence alignment. Multiple sequence alignment of (A) nsp1a/1 and (B) nsp1a/2 proteins sourced from 10 different mamastroviruses (MA). Sites of signal peptidase-mediated cleavage between nsp1a/1 and nsp1a/2 were predicted using SignalP6.0. Sequences were aligned using MUSCLE and visualized with Jalview. Residue conservation among isolates is highlighted from white (low) to dark blue (high). (*) MA01/HAstV1 sequence, (**) MA09/VA1 sequence.
(TIF)

S1 Movie HAstV1-driven ER remodeling by long-term, time-lapse imaging. Compiled frames from live-cell imaging of HAstV1 infected (MOI = 3) Huh7 cells expressing the mCherry-KDEL ER marker (gray). Images were acquired every 20 minutes from 00:00–20:00 hpi. Scale bars represent 20 µm.
(AVI)

S2 Movie Dynamics of typical ER morphology by long-term, time-lapse imaging. Compiled frames from live-cell imaging of mock-treated Huh7 cells expressing the mCherry-KDEL ER marker (gray). Images were acquired every 20 minutes from 00:00–20:00 hpi. Scale bars represent 20 µm.
(AVI)

S3 Movie Recruitment of peripheral ER to condensed aggregates. Compiled frames from a higher magnification of S1 Movie representing images taken between 14:20 and 18:00 hpi. Smaller particles of condensing ER undergoing recruitment to the larger aggregate are highlighted in cyan.
(AVI)

S1 Table Primer sequences used in this study. (DOCX)

S1 Data Excel tables include all values used to generate graphs. (XLSX)
