# Supplementary figures and images for: Endoplasmic reticulum-anchored nonstructural proteins drive human astrovirus replication organelle formation
Source: PLoS Pathog. 2025 Sep 22;21(9):e1013538. doi: 10.1371/journal.ppat.1013538 (PMC12469148; doi:10.1371/journal.ppat.1013538)

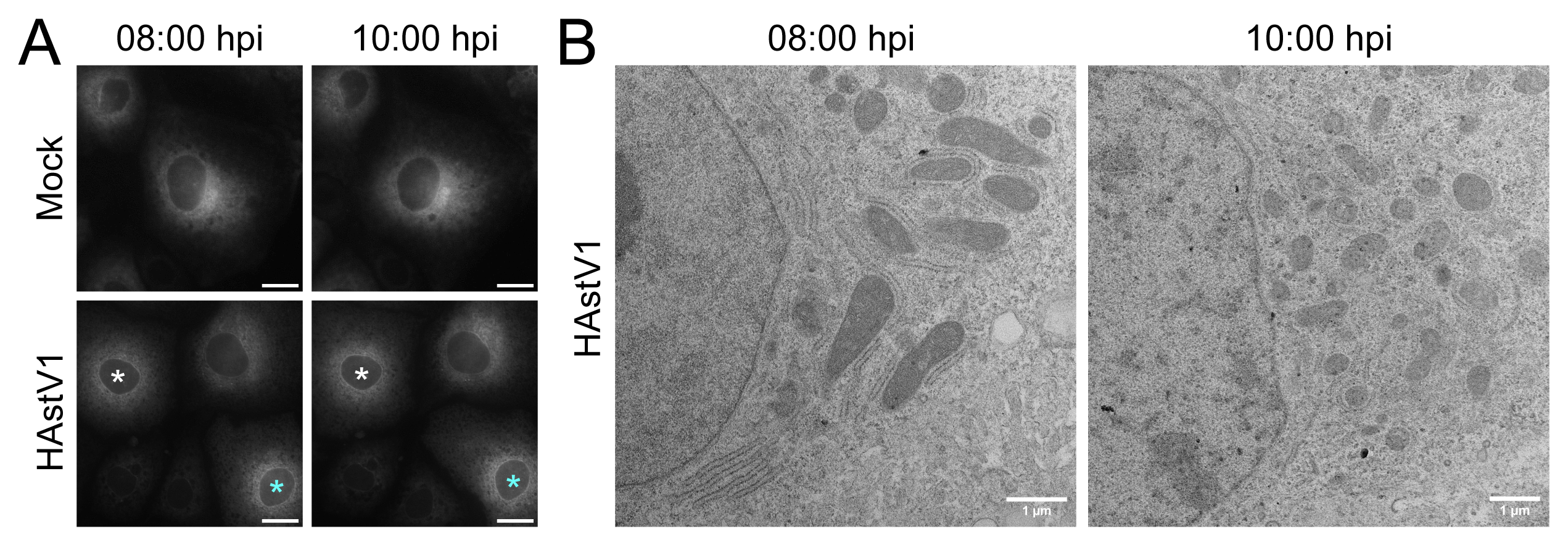

Supplement: S1 Fig — (A) Still frames from live-cell imaging of mock or HAstV1 infected (MOI = 3) Huh7 cells expressing the mCherry-KDEL ER marker (gray) at 8 and 10 hours post infection (hpi). Infected cells are indicated by an asterisk (*), with cyan indicating the cell highlighted in Fig 2C and S3 Movie. Scale bars represent 20 µm. (B) Transmission electron microscopy (TEM) of sectioned HAstV1 infected (MOI = 3) Huh7 cells fixed at 8 and 10 hpi. Scale bars represent 1 µm. (TIF) [file ppat.1013538.s001.tif]

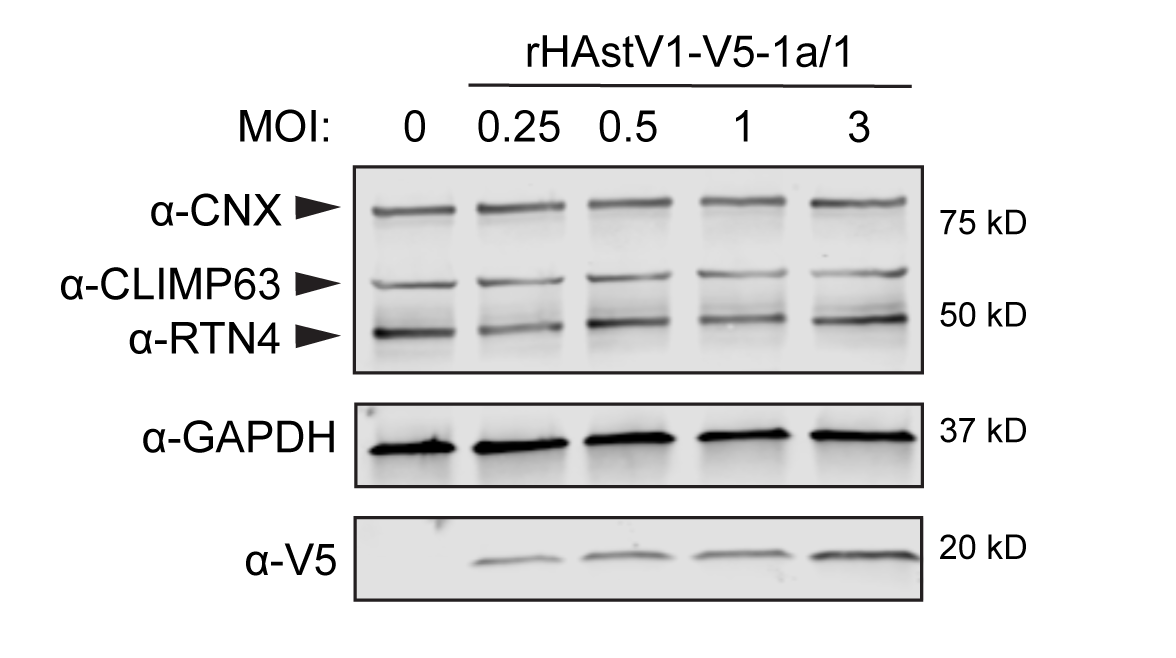

Supplement: S2 Fig — Immunoblot of mock or rHAstV1-V5-1a/1 infected (MOI = 0.25, 0.5, 1, 3) Caco2 cells lysed at 24 hpi. Immunoblots were probed for ER resident proteins (calnexin, CLIMP63, RTN4), V5, and GAPDH. (TIF) [file ppat.1013538.s002.tif]

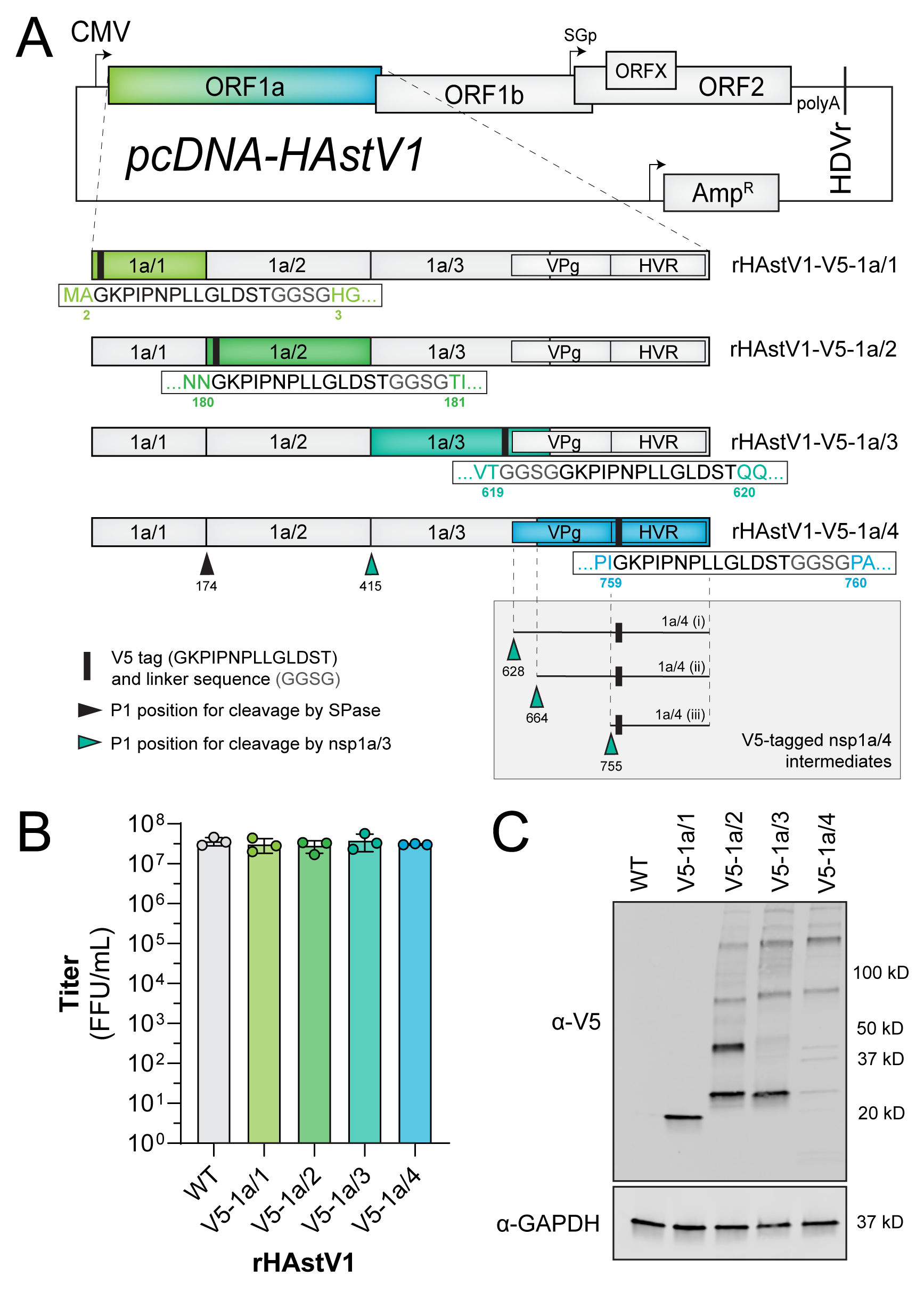

Supplement: S3 Fig — (A) Schematic of the pcDNA-HAstV1 infectious clone with V5-epitope tag locations within nsp1a/1 (lime), nsp1a/2 (green), nsp1a/3 (teal), and nsp1a/4 (blue) indicated by black rectangles with the specific inserted sequences and amino acid positions displayed below the tag. The P1 position of junctions targeted for cleavage by host signal peptidase (SPase, black) and nsp1a/3 (teal) is shown as arrowheads with the indicated P1 amino acid positions. (B) Titers of WT and rHAstV1-V5 viruses recovered from pcDNA-HAstV1 transfected Huh7 cells. Data are shown as the average ± SD focus-forming units per milliliter (FFU/mL) of supernatants serially diluted on Caco2 cells. (C) Immunoblot of lysates from WT or rHAstV1-V5 infected (MOI = 3; 24hpi) Caco2 cells. Immunoblots were probed for V5 and GAPDH. (TIF) [file ppat.1013538.s003.tif]

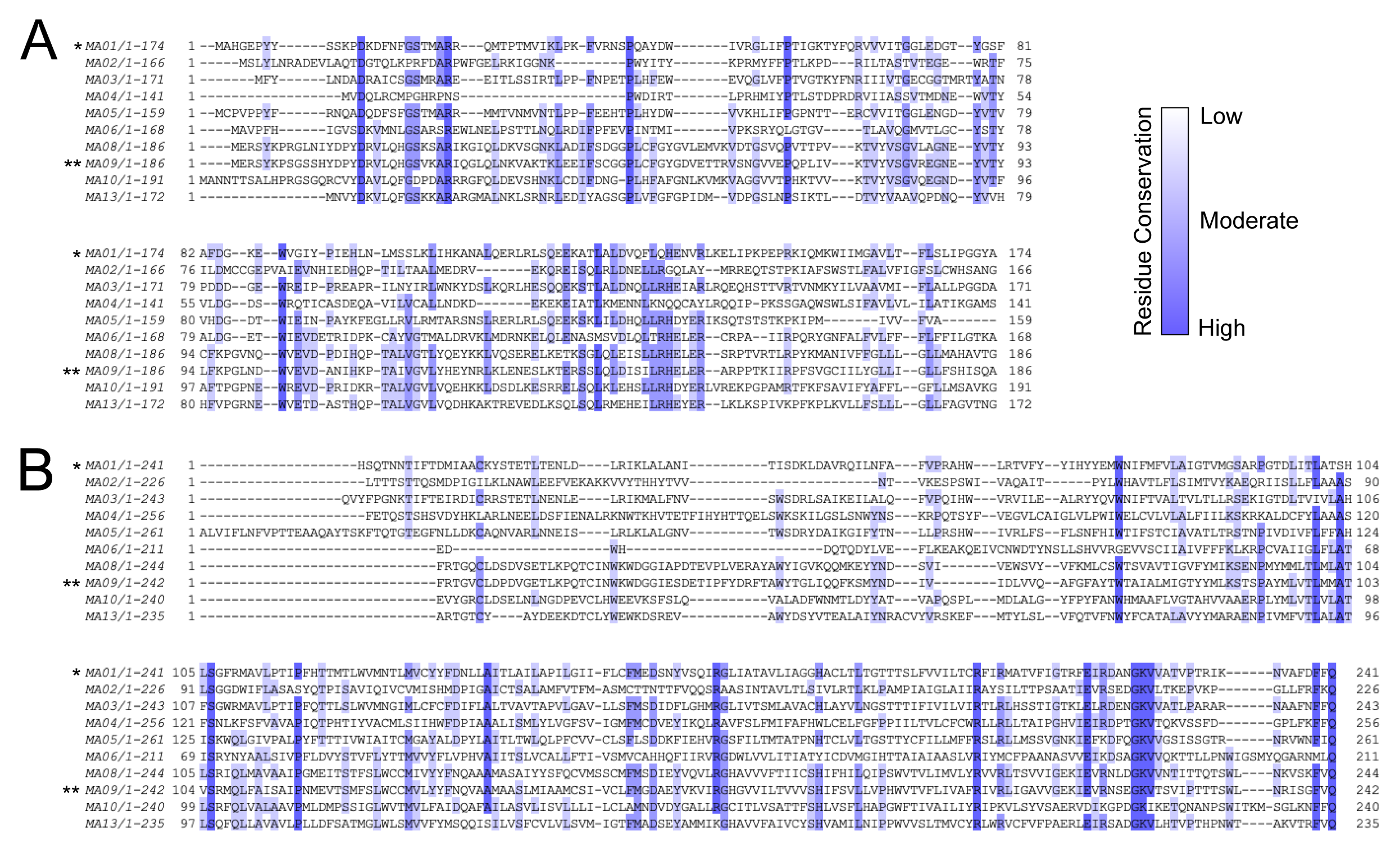

Supplement: S4 Fig — Multiple sequence alignment of (A) nsp1a/1 and (B) nsp1a/2 proteins sourced from 10 different mamastroviruses (MA). Sites of signal peptidase-mediated cleavage between nsp1a/1 and nsp1a/2 were predicted using SignalP6.0. Sequences were aligned using MUSCLE and visualized with Jalview. Residue conservation among isolates is highlighted from white (low) to dark blue (high). (*) MA01/HAstV1 sequence, (**) MA09/VA1 sequence. (TIF) [file ppat.1013538.s004.tif]
