# Supplementary material for: Endoplasmic reticulum-anchored nonstructural proteins drive human astrovirus replication organelle formation
Source: PLoS Pathog. 2025 Sep 22;21(9):e1013538. doi: 10.1371/journal.ppat.1013538 (PMC12469148; doi:10.1371/journal.ppat.1013538)
Supplement: S1 Table — (DOCX) [file ppat.1013538.s008.docx]

**Supplemental Table 1.** Primers used for PCR amplification.

| Name | Sequence (5’ to 3’) |
| --- | --- |
| AstV-PstI_F | gcgcaacgttgttgccattgctgca |
| AstV-AgeI_R | ctatttcatctgccttagtcaac |
| V5-1a/1_R | tcgagaccgaggagagggttagggataggcttacctgccatcttgttgttaa  ttgtatgg |
| V5-1a/1_F | ccctctcctcggtctcgattctacgggaggctctggtgcacacggtgagccata  ctatag |
| V5-1a/2_R | atcgagaccgaggagagggttagggataggcttaccgttgttggtctggc  tgtgcgc |
| V5-1a/2_F | ccctctcctcggtctcgattctacgggaggctctggtaccatatttactgacatg  atag |
| V5-1a/3_R | agaccgaggagagggttagggataggcttaccgcctcctgtaacaacagtcg  ttacgttc |
| V5-1a/3_F | ccctctcctcggtctcgattctacgggaggccaacaacctagtgttgcacta  gaacag |
| V5-1a/4_R | tcgagaccgaggagagggttagggataggcttaccaattggttttgcttggt  caaag |
| V5-1a/4_F | ctctcctcggtctcgattctacgggaggctctggtcctgccccgagaacaacca  agcc |
| HAstV1-1a/1_F | gcttggtaccgagctcggatccatggcacacggtgagcc |
| HAstV1-1a/1_R | gaattccaccacactggaggtgttgttggtctggctg |
| HAstV1-1a/2_F | gcttggtaccgagctcggatcccacagccagaccaacaacacc |
| HAstV1-1a/2_R | gaattccaccacactggactggaagaagtcaaatgcaac |
| VA1-1a/1_F | gcttggtaccgagctcggatccgagcgctcatacaagcctagt |
| VA1-1a/1_R | gaattccaccacactggaaagacacacaccagtcctaaa |
| VA1-1a/2_F | gcttggtaccgagctcggatcctttaggactggtgtgtgtctt |
| VA1-1a/2_R | gaattccaccacactggattgggtaaatttggactgaac |
| MluI-CMV_F | tacgggccagatatacgcgttgacattgattattgactag |
| SpBIP-GFP_R | cagcagcagcatcgcggccaccagggagagcttcatggcaagcttaagtttaaacgc |
| SpBIP-GFP_F | gcgatgctgctgctgctcagcgcggcgcgggccgtgagcaagggcgaggagctg |
